# Screening and Identification of Thermotolerant and Osmotolerant *Bacillus amyloliquefaciens* BKHE Isolated from Kinema of Eastern Nepal for Alkaline Protease Production

**DOI:** 10.1155/2022/6831092

**Published:** 2022-12-06

**Authors:** Gyanu Raj Pandey, Asmita Shrestha, Tika B. Karki, Shardul Neupane, Sabnam Ojha, Prashanna Koirala, Parash Mani Timilsina

**Affiliations:** ^1^Biotechnological Research and Development Center Pvt. Ltd, Bharatpur, Nepal; ^2^Shubham Biotech Nepal Pvt. Ltd, Bharatpur, Nepal; ^3^Department of Biotechnology, Kathmandu University, Dhulikhel, Nepal

## Abstract

Alkaline protease is one of the most important industrial enzymes which are excessively used in the detergent industry, food industry, feed industry, pharmaceutical industry, leather industry, etc. 60% of the produced alkaline protease is consumed by the detergent industry alone. In the present study, bacterial isolates that can produce alkaline protease for purpose of bio-detergent were screened among the isolates isolated from kinema (an alkaline fermented food of eastern Nepal). Selected bacterial isolates were further screened for hemolysis activity and the production of other hydrolytic enzymes. Four bacterial isolates selected were tested for their capacity to produce alkaline protease in five different fermentation mediums. Isolate BKHE produces a high amount of alkaline protease (0.4705 ± 0.035 U/mL/min) in fermentation medium M2 (sucrose, 11 g/L; yeast extract, 5 g/L; and KNO3, 5.2 g/l, pH 9). The selected isolate was identified as *Bacillus amyloliquefaciens* BKHE based on 16S rRNA sequencing and phenotypic features. This bacterial strain was also found to be thermotolerant (confluent growth at 50°C) and salt tolerant up to 10% NaCl concentration. With its versatile ability, bacterial isolate or purified enzymes have potential applications in the food and detergent industry.

## 1. Introduction

During the 20th century, also called the age of organic chemistry, coastal environments receive a variety of land-derived organic inputs, both natural and synthetic. Among them, detergents are probably the largest class of technical products for domestic use [1]. Then, with the rise of enzyme technology, the field of detergent science has been more advanced and the market expanded. Enzymes with a variety of applications in the detergent industry, food industry, feed industry, pharmaceutical industry, leather industry, peptide synthesis, and recovery of silver from used X-ray films are developed [2]. Among these, detergent industries are the primary consumers of enzymes, in terms of both volume and value [3], consuming about 60% of all the enzymes produced [4]. Among hydrolytic enzymes, microbial alkaline protease dominates commercial applications with a significant share of the market captured by subtilisins and/or alkaline protease from *Bacillus* spp. for laundry detergent applications [5]. Alkaline proteases added to laundry detergents enable the release of proteinaceous material from stains [6]. The increased usage of these proteases as the detergent additive is mainly due to the cleaning capabilities of these enzymes in environmentally acceptable nonphosphate detergents [2]. Along with alkaline protease; cellulase, lipase, and amylase are the major enzymes incorporated in detergent formulation, amylase being the second most important enzyme used in the detergent industry [7].

Although protease-producing microorganisms, plants, and animals are widespread, microbial communities are preferred due to their growth and simplicity for the generation of new recombinant enzymes with desired properties [8]. Protease-producing microbes and their various applications were reviewed elsewhere [2]. Especially, there are many studies on the production of protease from the genus *Bacillus* [9–14], and many studies claim that alkaline protease produced by *Bacillus* spp. can remove blood stains and blood clots [8, 15–17].

*Bacillus* species are mostly isolated from soil, or from environments that may have been contaminated directly or indirectly by soil, but are also found in water, food, and clinical specimens. This group of bacteria exhibits a wide diversity of physiological abilities, ranging from psychrophilic to thermophilic, and acidophilic to alkaliphilic; some strains are salt tolerant and some are halophilic [18]. It is important to select an isolation source for isolating bacterial species with proper physiological features [19].

Our source of isolation is kinema, a soybean-fermented food, which is traditionally consumed by the non-Brahmin Nepalese inhabiting the hilly region of eastern Nepal, Darjeeling hills, and Sikkim of India, and some parts of Bhutan [20]. The process of making kinema is discussed elsewhere [21]. The desired state of fermentation is indicated by the formation of a typical kinema flavor dominated by ammonia [21], so kinema is mostly alkaline and the pH range is from 6.6 to 8.5 [20]. Different *Bacillus* spp. have been previously reported from kinema from a different region of India [22–26]. Screening and production of alkaline protease for bio-detergent application from kinema samples was not reported to date. We have selected kinema as it is already a selective niche for *Bacillus* with alkaline pH. We aim to isolate and identify thermotolerant and osmotolerant *Bacillus* sp. that are capable of producing alkaline protease.

## 2. Materials and Methods

### 2.1. Microorganisms and Their Maintenance

The organisms used in this study were isolated from kinema samples on nutrient agar. Three sundried kinema samples were collected from the hilly area of the Dhankuta District of Nepal. 0.2 gm of kinema samples were homogenized with 10 mL of sterile physiological saline (0.85%). The kinema homogenate was taken for serial dilution and spread plated on nutrient agar followed by incubation at 37°C for 24 hours under aerobic conditions. Isolates having different colony morphology were collected and purified by sub-culturing in a nutrient agar plate. Among the isolates, Gram-positive and rod-shaped bacteria were taken for further research activities.

All the isolated organisms were preserved by lyophilization [27] and stored at −80°C for long-term storage and a bacterial slant was prepared for research activities.

### 2.2. Alkaline Protease Screening

Ten gram-positive and rod-shaped bacterial isolates were screened on an alkaline agar medium with sodium caseinate for their ability to produce alkaline protease (0.5% tryptone, 0.25% yeast extract, 0.1% glucose, 1% sodium caseinate, 0.44% trisodium citrate. 2H_2_O, 20 mM CaCl_2_. 6H_2_O, and 1.5% agar (pH: 8)) [10, 14]. They were incubated at 30°C for 48 hours [14].

### 2.3. Screening for Hemolysis Activity

To the sterile blood agar base which has been melted and cooled to 45 to 50°C, 5% (vol/vol) sterile defibrinated blood that has been warmed to room temperature was added. The media then was poured into Petri plates avoiding any bubbles. They were incubated at 37°C for 24 hours. Hemolysis was recorded by the appearance of a zone of clearing around the colonies [28].

### 2.4. Screening for Production of Hydrolytic Enzymes

An amylolytic test was carried out by culturing bacteria on starch agar (Nutrient Agar with 0.2% starch), incubated at 37°C. After 48 hours the plates were flooded with 1% Lugol's iodine reagent for 10 mins and drained off. The clear halo region indicated starch hydrolysis [18].

The bacteria were grown on nutrient agar with 1% CMC for cellulolytic activity. The CMC agar plates were incubated at 37°C for 24 hours. At the end of the incubation, the agar medium was flooded with an aqueous solution of Congo red (1% w/v) for 15 minutes. The Congo red solution was then poured off, and the plates were further treated by flooding with 1 M NaCl for 15 minutes. The formation of a clear zone of hydrolysis indicated cellulose degradation [29].

The ability of lipase production was carried out in Petri dishes using a medium (composition: peptone 0.5%; yeast extract 0.3%; tributyrin 1% and agar 2% in distilled water) [30]. The cultured plates were incubated at 30°C for 48 hours.

Skim milk powder, 5 g in 50 ml of distilled water; agar, and 1 g in 50 ml of distilled water were autoclaved separately at 121°C, cooled to 45°C, mixed, and poured into Petri plates. The cultured plates were incubated at 30°C for 48 hours. The halo region indicated protease production [18].

Gelatin liquefaction (the formation of a liquid) was tested by stabbing gelatin agar (semisolid with 7.5 g/L agar) in deep tubes. After 48 h of incubation, the cultures were placed in a refrigerator at 4°C until the bottom resolidifies. If gelatin was hydrolyzed, the medium will remain liquid after refrigeration. If gelatin was not hydrolyzed, the medium will resolidify during the time it is in the refrigerator [31].

### 2.5. Alkaline Protease Production in Different Medium Composition

The selected isolates were cultured in different production mediums: **M1** (gelatin broth containing: gelatin, 10 g/L; casein enzymatic hydrolyzate, 10 g/L; and NaCl (w/v), 100 g/L; pH 9) [11]; **M2** containing ((g/L): sucrose, 11; yeast extract, 5; and KNO3, 5.2, pH9, modified with no trace elements and optimal concentration of given composition) [32]; **M3** containing (1% glucose, 0.5% peptone, 0.5% yeast extract, 0.5% KH2PO4, 20% NaCl, 20% Na_2_CO_3_ autoclaved separately and final pH 10.0) [12]; **M4** containing ((g/L): casein, 5; peptone, 5; yeast extract, 2; NaCl, 5; MgSO_4_.7H_2_O, 0.2; CaCl_2_, 0.1; K_2_HPO_4_, 1; and Na_2_CO_3_, 10, pH 10.3. Sodium carbonate was separately autoclaved and added to the rest of the medium after cooling) [33] and **M5** containing ((g/L): glucose, 10; peptone, 5; yeast extract, 5; K_2_HPO_4_, 1; MgSO_4_, 7; H_2_O, 0.2; Na_2_CO_3_, 10; NaCl, 5; pH 10.5) [13]. All the experiments were carried out at 37°C, 120 rpm, and 48 hours of incubation.

### 2.6. Protease Assay

The reaction mixture in a total volume of 11 ml was composed of 5 ml of 0.65% casein in 50 mM Potassium Phosphate buffer, pH 7.5, and an enzyme solution of 1 ml, 0.75 ml, and 0.5 ml for 3 different test sets. After 10 min incubation at 37°C, the reaction was terminated with 5 ml of 110 Mm trichloroacetic acid followed by another incubation of 30 min at 37°C. To 2 ml test filtrate, 5 ml of 500 mM sodium carbonate was added followed by 1 ml 1 M Folin-Ciocalteu's phenol reagent. The reaction mixture was incubated at 37°C for 30 min and then allowed to cool to room temperature and test filtrate absorbance measured at 660 nm along with blank and standard solution in UV-1800 Shimadzu spectrophotometer. One unit of protease activity was defined as the amount of enzyme which released 1 *μ*mole tyrosine per min under the assay condition [34].

### 2.7. Identification of Selected Bacterial Isolate

#### 2.7.1. Molecular Characterization of Selected Isolate

DNA extraction and Sequencing of 16S rRNA Gene: bacterial isolate BKHE was cultured in 10 mL nutrient broth. Incubation was performed at 37°C for 24 hours. The genomic DNA of the bacteria was isolated according to the procedure of Sambrook and Russel [35].

The 16S rRNA gene of the bacterial isolate was amplified using universal primers FL (5′-AGAGTTTGATCMTGGCTCAG-3′) and 1492R (5′-TACGGYTACCTTGTTACGACTT-3′) [36]. The amplified segment was purified and sequenced at Macrogen Inc., South Korea using sequencing primers 785F (5′-GGATTAGATACCATGGTA-3′) and 907R (5′-CCG TCAATTCMTTTRAGTTT-3′).

Raw sequences were assembled and trimmed using the codon code aligner. The contig sequence generated was subjected to BLASTN and the database “rRNA/ITS databases” was selected. Twelve highly similar sequences (based on blast results) were taken in FASTA format for phylogenetic analysis.

#### 2.7.2. Maximum Parsimony Analysis of Taxa

The evolutionary history was inferred using the maximum parsimony method. The bootstrap consensus tree inferred from 1000 replicates is taken to represent the evolutionary history of the taxa analyzed [37]. The MP tree was obtained using the tree-bisection-regrafting (TBR) algorithm [38] with search level 1 in which the initial trees were obtained by the random addition of sequences (10 replicates). This analysis involved 13 nucleotide sequences. All positions with less than 95% site coverage were eliminated, i.e., fewer than 5% alignment gaps, missing data, and ambiguous bases were allowed at any position (partial deletion option). Evolutionary analysis was conducted in MEGA X [39].

#### 2.7.3. Morphological and Biochemical Characterization

The selected protease-producing isolate BKHE was identified using morphological and biochemical characteristics, according to -Bergey's Manual of Systemic Bacteriology [18] while the sugar utilization test was performed as manufacturer's instruction using Himedia KB009TM HiCarbo Kit (KB009A/KB009B1/KB009C).

### 2.8. Salt and Temperature Tolerance Test for Selected Strain

For temperature tolerance, the organism was cultured in Nutrient broth and incubated at different temperatures (30°C, 40°C, 45°C, 50°C, 60°C) and for salt tolerance, the organism was cultured in Nutrient broth with different salt concentrations (2%, 6% and 10% (w/v)) [18]. The cell density was measured using a DEN-1B Grant bio Densitometer. 18 phi test tubes were used for generating data and data were obtained in McFarland standards.

### 2.9. Statistical Analysis

Data analysis was performed using OriginPro 9.0, IBM SPSS 15.0, and Microsoft excel. ANOVA was performed for quantitative data where the Tukey test was used to compare the means at a 95% confidence interval.

## 3. Result

### 3.1. Isolation and Screening

**Figure 1:**
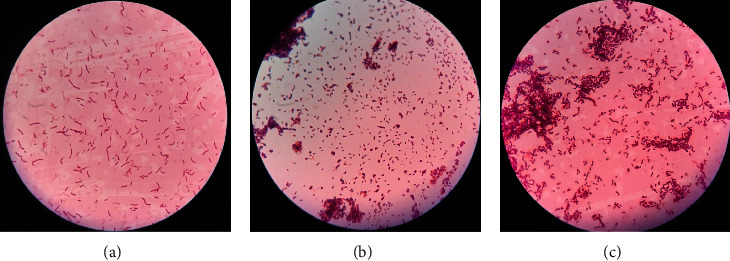
Gram-positive microscopic observation of some isolates (1000X): (a) isolate BKTD; (b) isolate BKHA; (c) isolate BKHE.

Among the purified isolates, only 10 isolates were found to be Gram-positive and rod-shaped bacteria. Only Gram-positive and rod-shaped bacteria were further screened for alkaline protease activity.  Figure 1 illustrated three of the Gram-positive and rod-shaped bacteria.

### 3.2. Screening for Alkaline Protease Positive Isolates

**Figure 2:**
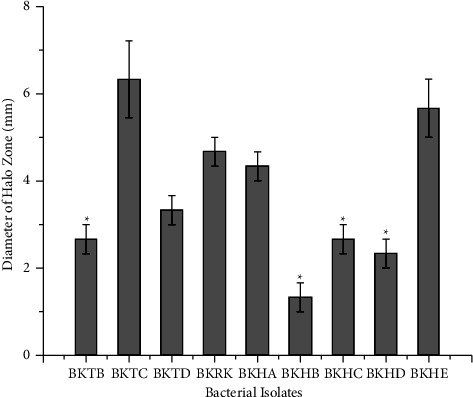
Comparison of halo zones in sodium caseinate agar by different bacterial isolates. ^*∗*^The mean diameter of the halo zone is significantly lower in comparison with the mean value of BKTC at *α* = 0.05.

Among nine selected isolates studied for casein hydrolysis, isolates BKTB, BKHB, BKHC, and BKHD were found to produce significantly smaller halo zone in comparison with isolate BKTC (6.33 mm, SE = 0.88) at *p* < 0.05 confidence level. The differences in the diameter of halo zones of isolates BKTD (3.33 mm, SE = 0.33), BKRK (4.66 mm, SE = 0.33), BKHA (4.33 mm, SE = 0.33), and BKHE (5.66 mm, SE = 0.67) were insignificant at *p* < 0.05 confidence level as shown in Figure 2. One of the selected isolates (BKTA) showed a negative result in casein hydrolysis.

### 3.3. Screening for Hemolytic Activity and Production of Hydrolytic Enzymes

**Figure 3:**
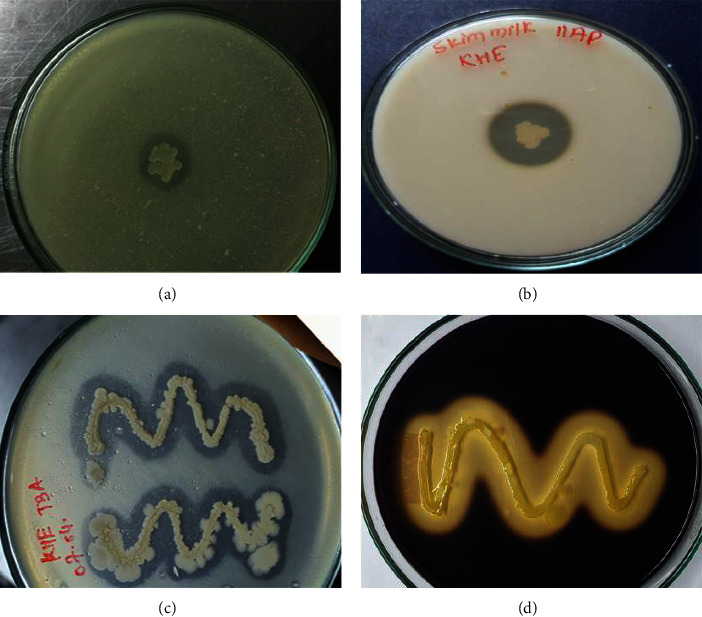
Hydrolytic enzymes production test for isolate BKHE: (a) alkaline protease screening in sodium caseinate agar (pH: 8); (b) protease screening in skim milk agar; (c) lipase screening in tributyrin agar; (d) amylase screening in starch agar.

Further screening of bacterial isolate was based on its capabilities to hydrolyze blood cells and produce other hydrolytic enzymes. Results are shown in Table 1, isolates BKTB, BKTC, BKTD, BKRK, and BKHE were found to show *β*-hemolysis. Along with hemolysis property, these isolates were able to produce other hydrolytic enzymes which are widely used in detergent formulation after protease.  Figure 3 illustrated the hydrolytic enzymes produced by one of the isolated BKHE.

### 3.4. Alkaline protease production in different medium compositions

**Figure 4:**
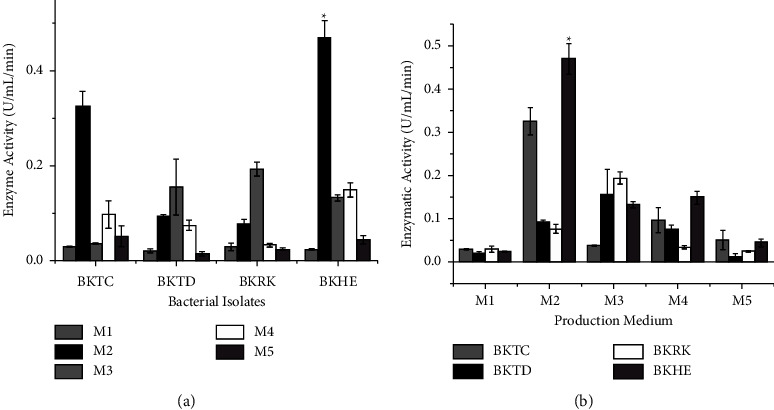
Comparison of enzyme alkaline protease activity produced by different bacterial isolates (a) and in different production mediums (b).

The one-waybetween-groups analysis was performed to observe the enzymatic activity of different bacterial isolates (Figure 4(a)), and Figure 4(b) illustrated the enzymatic activity in different production mediums. While comparing means of enzymatic activity between different medium compositions produced by different bacterial strains, cell-free fermentation broth of isolates BKHE (0.4705 U/mL/min, SE = 0.035) and BKTC (0.3252 U/mL/min, SE = 0.031) showed significantly high enzymatic activity in production medium M2 at *p* < 0.05 confidence level; cell-free fermentation broth of BKTD (0.155 U/mL/min, SE = 0.059) and BKRK (0.193 U/mL/min, SE = 0.015) showed significantly high enzymatic activity in production medium M3 at *p* < 0.05 confidence level, as shown in Figure 4(a).

But while comparing means of enzymatic activity between different bacterial isolates, cell-free fermentation broth of isolate BKHE in production medium M2 showed significantly high enzymatic activity than other bacterial isolates at *p* < 0.05 confidence level. So, a high enzyme-producing isolate was found to be isolate BKHE and the best production medium among the compared ones was production medium M2.

### 3.5. Identification of Selected Bacteria

#### 3.5.1. Molecular Characterization of Selected Isolate

**Figure 5:**
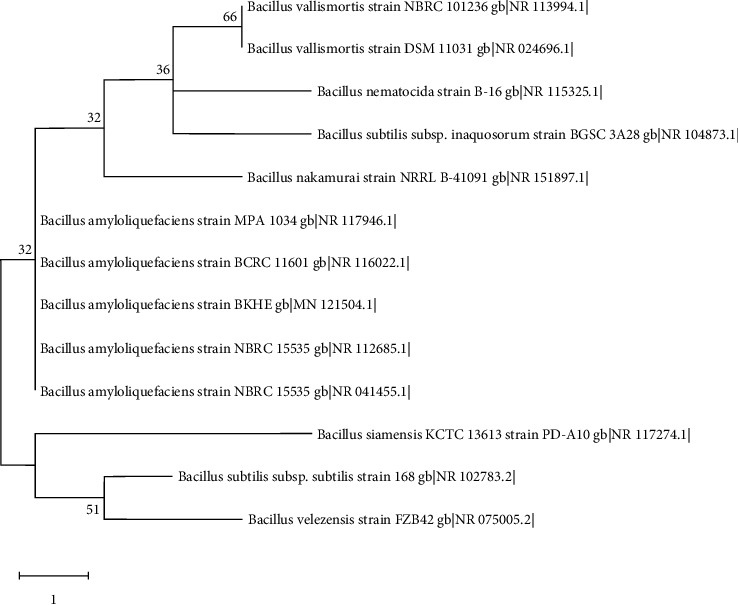
Maximum parsimony tree generated from 16S rRNA sequence data. Branches corresponding to partitions reproduced in less than 50% of bootstrap replicates are collapsed. The percentage of replicate trees in which the associated taxa clustered together in the bootstrap test is shown next to branches.

Based on 16S rRNA gene sequence analysis, isolate BKHE showed high sequence similarity to members of the genus *Bacillus* (Figure 5). BLAST result among “16S ribosomal RNA sequences (Bacteria and Archaea)” database BKHE showed 99.93% similarity with *Bacillus amyloliquefaciens* strain NBRC 15535; 99.86% similarity with *B. amyloliquefaciens* strain BCRC 11601; 99.59% similarity with *B. velezensis* strain FZB42 and *B. valismortis* strain DSM 11031; 99.52% similarity with *B. nematocida* strain B-16, *B. subtilis* subtilis strain 168, and *B. nakamurai* strain NRRL B-41091.

#### 3.5.2. Phenotypic Description of *B. amyloliquefaciens* BKHE

Gram-stain-positive rods of size 3–5 *μ*m, motile, thermotolerant, and subterminal ellipsoidal spore-forming. Colonies on nutrient agar medium were dirty white, smooth, slightly viscous, slightly raised and circular, and approximately 3-4 mm in diameter at 37°C after 24 h. Catalase positive but oxidase negative. Positive for acetoin, gelatinase, lipase, proteinase, and amylase production but negative for indole, H2S, urease, cellulose, and arginine dihydrolase. Nitrate was reduced to nitrite. Citrate was utilized but not malonate. It was able to utilize maltose, fructose, glucose, trehalose, sucrose, glycerol, sorbitol, mannitol, and cellobiose. It was sensitive to ciprofloxacin (30 mcf), gentamycin (10 mcf), tetracycline (30 mcf), moxifloxacin (5 mcf), levofloxacin (5 mcf), azithromycin (15 mcf), erythromycin (15 mcf), vancomycin (30 mcf), and rifampicin (5 mcf). Growth of up to 10% NaCl was reported. Confluent growth was reported up to 50°C. Confluent growth up to pH 9.5 and no growth at pH 4.5 was reported.

### 3.6. Salt Tolerance and Temperature Tolerance Test for Selected Strain

**Figure 6:**
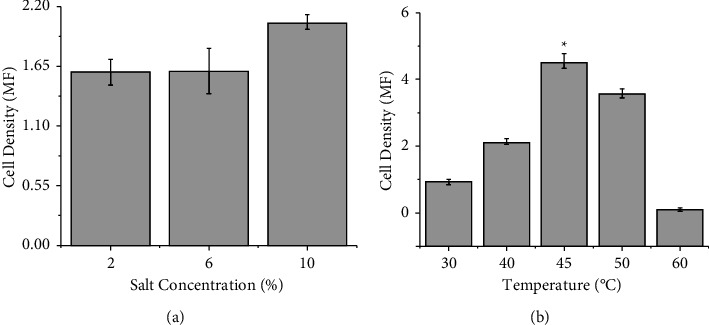
Comparison of cell density of *B. amyloliqueficiens* BKHE (a) in different salt concentrations and (b) at different temperatures (b).

Selected strain *B. amyloliqueficiens* BKHE was grown in different salt concentrations. Cell density was not significantly different among the medium with different salt concentrations at *p* < 0.05 confidence level as illustrated in Figure 6(a). Cell density at 10% sodium chloride concentration was 2.05 ± 0.07 MF. It was also cultured in nutrient broth and incubated at a different temperature, high cell density was observed at 45°C (4.55 MF, SE = 0.21) which is significantly higher than cell densities when incubated at 30°C, 40°C, 50°C, and 60°C at *p* < 0.05 confidence level as shown in Figure 6(b).

## 4. Discussion

We have identified alkaline protease, amylase, lipase, and surfactant-producing*B. amyloliquefaciens* strain BKHE, which was isolated from the kinema sample from eastern Nepal. Only isolate with viscous colony texture, Gram-positive and, rod-shaped were screened for alkaline protease production, as these are the basic features of *Bacillus* spp [18]. Among nine alkaline protease-positive strains, five of them (BKTB, BKTC, BKTD, BKRK, and BKHE) were able to hydrolyze blood cells. But the result of screening for alkaline protease showed that isolate BKTD produced a significantly smaller halo zone when compared with other hemolysis-positive isolates. So, only isolates BKTC, BKTD, BKRK, and BKHE were further compared quantitatively for the production of alkaline protease in different production mediums. A hemolytic activity might indicate the bacterial isolate is a biosurfactant producer, although other various lytic enzymes produced by strain may cause hemolysis [41]. Biosurfactants, along with their wide applications, are antimicrobials too [42]. So, the product from hemolytic bacteria along with protease activity can be effectively used in hospitals to remove blood spills and clots and kill infectious microbes. Another screening parameter was the capability to produce other hydrolytic enzymes. Since other enzymes such as amylase, lipase, and cellulase are also used in detergent formulations, so the capacity to produce these will be of great significance. Among four selected isolates, BKTC and BKTD were found to produce all sets of hydrolytic enzymes, while BKHE and BKRK were cellulase negative (from Table 1). Since the production cost and downstream process cost of the enzyme are high, it is important to reduce the cost to make these enzymes sellable to detergent industries. The benefit of producing multiple hydrolytic enzymes lies here, as multiple enzymes can be coproduced in the same fermentation media by the same bacterial strain. As these enzymes are used together not only in detergent industries, these are used together in the food and pharmaceutical industries too. Coproduction of cocktail enzymes is one way to reduce the cost of production and these approaches were found to be practical by other researchers [4, 7, 43].

Among the four isolates selected, the highest alkaline protease was produced by BKHE in medium M2 (sucrose, 11 g/L; yeast extract, 5 g/L; and KNO3, 5.2 g/l, pH 9). While comparing different media compositions, M2 was found to be best among the medium compared. BKTC also showed high production (significantly high when compared with BKTD and BKRK but significantly low when compared with BKHE at a confidence level of *p* < 0.05) and this paper only deals with the preliminary screening and media selection for the production of alkaline protease. Optimum conditions are yet to be identified for the production of alkaline protease production. The optimum condition depends on temperature, pH, media composition, oxygen transfer rate, and different bacterial strains [13, 32, 44–49]. So, there are many factors to be considered for optimal production from the selected isolate. In our study, medium M2 has been identified as the best medium among the selected ones for protease production. Sucrose in the medium induces protease production [32] and nitrate salt helps to accelerate the production [50]. While in some, yeast extract gave high protease production than nitrate [13], and in some gelatin induced protease production [11]. There has also been a report of better alkaline production in the presence of glucose as a carbon source and yeast extract and peptone as an organic nitrogen source [12, 33]. The results shown by our isolates are also different for different isolates. BKHE produced high alkaline protease in presence of yeast extract, sucrose, and potassium nitrate and low production in gelatin-based and glucose-based mediums. So, the selection of appropriate carbon and nitrogen source is highly dependent upon the bacterial strain and needs to be optimized as per the requirement of the strain.

16S rRNA sequencing showed that the isolate is “*Bacillus amyloliquefaciens* BKHE” and further phenotypic features of the strain were studied to conclude the taxonomy with the polyphasic approach [18, 51]. Phenotypic features of the selected strain were compared with phenotypic features of *B. amyloliquefaciens* [18] *B. velezensis* [40] and *B. valismortis* [18] obtained from the database. Since BLAST result and phylogenetic analysis (Figure 6) suggest the strain belongs to *B. amyloliquefaciens* but differentiating margins with other species were little. While comparing the phenotypic features in Table 2, BKHE was found to match most of the features with *B. amyloliqueficiens*, the only differences observed were in the fermentation of raffinose and salicin. *B. amyloliqueficiens* and BKHE are oxidases negative while *B. velezensis* and *B. valismortis* are positive. Phenotypic features of BKHE that differ from *B. velezensis* were H2S production and utilization of lactose, xylose, raffinose, melibiose, L-arabinose, mannose, salicin, inositol, *α*-methyl-D-glucoside, and ONPG as shown in the table. Differences between BKHE and *B. valismortis* also can be observed in Table 2. On basis of both 16S rRNA sequencing and phenotypic features of the strain BKHE, it was identified as “*B. amyloliqueficiens* BKHE.”

Alkaline protease with desirable properties such as activity at high pH, salinity, and temperature is suitable for commercial application in detergent industries [44, 52]. Proteases from hyperthermophiles and thermophiles are the natural choice for exploring inherent heat stability [53]. So, exploring ecological niches such as extreme or high temperature, pH, salinity, pressure, and toxicity is one of the promising ways to isolate microbes with required properties [19, 53]. Kinema, also being alkaline and dried, makes a suitable niche for isolating alkaline protease-positive isolates. *B. amyloliquefaciens* BKHE was found to have confluent growth at 50°C as the kinema sample was sundried and thermotolerant strains were able to cope with the drying process. Also, this strain was found to be tolerant to high salt concentration and high pH, so alkaline protease produced from this isolate could be used in formulations of bio-detergent, although further screening in purified enzymes is yet to be done. Also, this strain could be used for the coproduction of protease, amylase, and lipase, which are also used in detergent formulations.

## 5. Conclusion

A bacterial isolate with high alkaline protease-producing ability was identified as *B. amyloliquefaciens* BKHE based on 16S rRNA sequencing and phenotypic features. The strain was found to be cellulase negative and was positive for protease, amylase, and lipase. Also, its ability to withstand high temperatures and osmolarity opens the door for versatile applications of bacteria or enzymes in the food, feed, and detergent industry. Since our strain produces multiple enzymes, it can be used for the coproduction of multiple enzymes which could be used in the detergent and food industry.

## Figures and Tables

**Table 1:** Results of hemolytic activity and hydrolytic enzymes.

Isolates	Amylase	Lipase	Cellulase	Gelatinase	Protease (skimmed milk)	Hemolysis
BKTB	−	+	−	+	+	+
BKTC	+	+	+	+	+	+
BKTD	+	+	+	+	+	+
BKRK	+	+	−	+	+	+
BKHA	−	+	+	−	+	−
BKHB	+	+	+	+	−	−
BKHC	+	+	+	+	+	−
BKHD	+	+	+	+	+	−
BKHE	+	+	−	+	+	+

**Table 2:** Phenotypic features of selected isolate and comparison with closely related species based on BLAST result.

Tests	BKHE	*B. amyloliqueficiens* [18]	*B. velezensis* [40]	*B. valismortis* [18]
Colony characteristics				
Colony form	Circular			
Elevation	Slightly raised			
Margin	Undulate			
Texture	Slightly viscous			
Opacity	Opaque			
Surface	Smooth			
Chromogenesis	Dirty white		Creamy white	
Diameter (cm)	0.3–0.4			
Gram staining	+	+	+	+
Shape of cell	Rod	Rod	Rod	Rod
Size of cell	3–5 um		1.5–3.5 um	
Spore staining	+	+	+	+
Spore position	Subterminal			
Spore shape	Ellipsoidal	Ellipsoidal	Ellipsoidal	Ellipsoidal
Growth at temperature				
30	+		+	+
40	+	+	+	+
45	+		+	
50	+	±	−	+
60	−	−	−	
Nacl tolerance				
1%	+		+	
2%	+	+	+	+
5%	+	+	+	+
Growth at Ph				
4.5	−	±		
6	+	+	+	+
7.2	+	+	+	+
8	+	+	+	
9.5	+	+	+	
Indole production	−	−	−	
Voges-Proskauer	+	+	+	+
Hydrogen sulfide	−		+	
Arginine dihydrolase	−	−	−	
Motility	+	+	+	+
Nitrate reduction test	+	±	+	+
Catalase	+		+	+
Oxidase	−	−	+	+
Citrate utilization	+	±		+
Malonate utilization	−	−		
Acid from				
Lactose	−	±	+	−
Xylose	−	±	+	+
Maltose	+	+	+	+
Fructose	+	+	+	+
Dextrose	+	+	+	+
Galactose	−	−	−	+
Raffinose	−	+	+	
Trehalose	+	±	+	+
Melibiose	−		−	+
Sucrose	+	+	+	+
L-arabinose	−	±	+	+
Mannose	−	±	+	+
Inulin	−		−	
Sodium gluconate	−		−	
Glycerol	+	+	+	
Salicin	−	+	+	+
Dulcitol	−	−	−	
Inositol	−		+	
Sorbitol	+	±	+	+
Mannitol	+	+	+	+
Adonitol	−	−	−	
Arabitol	−		−	
Erythritol	−	−	−	
*α*-methyl-D-glucoside	−		+	
Rhamnose	−	−	−	+
Cellobiose	+	+	+	+
Melezitose	−		−	
*α*-methyl-D-mannoside	−		−	
Xylitol	−		−	
D-arabinose	−		−	
Sorbose	−		−	
Hydrolysis of				
Urea	−	−		−
Gelatin	+	+	+	
Lipid (tributyrin)	+			
Casein	+	+	+	+
Cellulose	−			
Starch	+	+	+	+
ONPG	−	±	+	
Esculin	+	+		
Resistance to antibiotics	AMP, CXM, AMC, CTX			
Sensitive to antibiotics	CIP, GEN, TE, MO, LE, AZM, E, VA,RIF			

AMP–ampicillin (10 mcf); CXM–cefuroxime (30 mcf); AMC–amoxicillin (10 mcf); CTX–cefotaxime (30 mcf); CIP–ciprofloxacin (30 mcf); GEN–gentamycin (10 mcf); TE–tetracycline (30 mcf); MO–moxifloxacin (5 mcf); LE–levofloxacin (5 mcf); AZM–azithromycin (15 mcf); E–erythromycin (15 mcf); VA–vancomycin (30 mcf); RIF–rifampicin (5 mcf).

## Data Availability

Data are available from https://www.ncbi.nlm.nih.gov/nuccore/MN121504
